# The heterogeneous wound microbiome varies with wound care pain, dressing type, and inflammatory gene expression

**DOI:** 10.1111/wrr.13184

**Published:** 2024-04-26

**Authors:** Amy Campbell, Jaewon Bae, Maria Hein, Stephen L. Hillis, Olivia N. Rebeck, Barbara A. Rakel, Elizabeth Grice, Sue E. Gardner

**Affiliations:** ^1^ The University of Iowa College of Nursing Iowa City Iowa USA

**Keywords:** microbiome, pain, wound dressing

## Abstract

Wound dressing changes are essential procedures for wound management. However, ~50% of patients experience severe pain during these procedures despite the availability of analgesic medications, indicating a need for novel therapeutics that address underlying causes of pain. Along with other clinical factors, wound pathogens and inflammatory immune responses have previously been implicated in wound pain. To test whether these factors could contribute to severe pain during wound dressing changes, we conducted an exploratory, cross‐sectional analysis of patient‐reported pain, inflammatory immune responses, and wound microbiome composition in 445 wounds at the time of a study dressing change. We profiled the bacterial composition of 406 wounds using 16S ribosomal RNA amplicon sequencing and quantified gene expression of 13 inflammatory markers in wound fluid using quantitative real‐time polymerase chain reaction (qPCR). Neither inflammatory gene expression nor clinically observed inflammation were associated with severe pain, but *Corynebacterium* and *Streptococcus* were of lower relative abundance in wounds of patients reporting severe pain than those reporting little or no pain. Wound microbiome composition differed by wound location, and correlated with six of the inflammatory markers, including complement receptor C5AR1, pro‐inflammatory cytokine interleukin (IL)1β, chemokine IL‐8, matrix metalloproteinase MMP2, and the antimicrobial peptide encoding cathelicidin antimicrobial peptide. Interestingly, we found a relationship between the wound microbiome and vacuum‐assisted wound closure (VAC). These findings identify preliminary, associative relationships between wound microbiota and host factors which motivate future investigation into the directional relationships between wound care pain, wound closure technologies, and the wound microbiome.

**Abbreviations**
ARG1: Arginase 1
ASV: amplicon sequence variant
CAMP: cathelicidin antimicrobial peptide
CLR: centred log ratio
CSSC: Clinical Signs and Symptoms Checklist
CT: cycle threshold
CWON: Certified Wound Ostomy Nurse
DMM: Dirichlet multinomial mixture
LCN2: Lipocalin 2
NMDS: non‐metric multidimensional scaling
NRS: numerical rating scale
qPCR: quantitative real‐time polymerase chain reaction
TNF‐α: tumour necrosis factor‐α
VAC: vacuum‐assisted closure
WCP: wound care procedures

## 1 INTRODUCTION

Wound care procedures (WCP), such as dressing changes, cause severe pain in as many as half of all patients.^1^, ^2^ Barrier tissues such as the skin are rich in nociceptors, sensory afferent neurons that induce protective responses from harmful and noxious stimuli.^3^, ^4^, ^5^ In skin and other peripheral tissues, these reflexive responses are perceived as pain. A severe pain event during a dressing change is stressful to both the patient and healthcare provider, and leads to anticipatory pain during subsequent WCP.^6^, ^7^, ^8^ Unfortunately, the mainstay pain control strategies, analgesic medications, and advanced dressings, are less than optimal in terms of effectiveness, side effects, and costs.^9^, ^10^ Thus, novel interventions to prevent severe pain during dressing changes are urgently needed.

Despite this need, the underlying causes of severe pain during dressing changes are incompletely understood. Wound factors such as shorter duration of injury, higher inflammation, acute wound aetiology, and higher resting wound pain have previously been linked to high levels of WCP pain.^1^, ^2^, ^11^ In addition to these clinical characteristics, however, the wound microbiota and inflammatory immune responses are also likely to contribute to pain during WCP, and offer promising targets for new pain mitigation strategies.

All wounds are colonised with microbiota upon breach of the skin barrier, which can consist of mixed communities of bacteria, fungi, and viruses. Depending on the context of tissue injury, these microbial communities mediate inflammation, host defence, wound repair, and even clinical outcomes.^12^ During infection, bacterial pathogens have been shown to interact with nociceptors to cause pain. *Staphylococcus aureus* produces pore‐forming toxins that directly activate nociceptors during infection to cause pain.^13^, ^14^ *Streptococcus pyogenes*, the causative agent of the intensely painful condition necrotizing fasciitis, induces pain directly through the toxin streptolysin S.^15^ Nociceptors express a variety of different receptors that recognise microbes, and can signal to immune cells by releasing neuropeptides.^16^ In turn, cytokines produced by immune cells, including interleukin (IL)‐1, can sensitise neurons and cause pain as well.^5^, ^17^

Here we hypothesized that the wound microbiota and local inflammation would be associated with pain during a wound dressing change. To test this hypothesis, we conducted an exploratory analysis of the microbiota of 406 wounds in a 445‐wound cohort which included chronic wounds such as pressure ulcers and venous ulcers as well as acute injuries such as surgical and traumatic wounds. In parallel, we assessed the local inflammatory response by measuring gene expression of 13 genes related to skin inflammation. Wound variables, including duration, depth, location, dressing types, clinical inflammatory signs, and wound type, were collected and analysed with respect to the microbiota and local inflammation.

We found that wound microbiota varied by wound aetiology and correlated with patient‐reported pain and local inflammatory gene expression. However, local inflammatory gene expression on its own was not associated with patient‐reported pain, despite a strong correlation with clinical signs of inflammation. Finally, we found that the type of wound dressing, and specifically, vacuum‐assisted wound closure (VAC) was related to the wound microbiota independently of wound aetiology or depth. These results provide further evidence that wound aetiology and other patient‐ and wound‐level factors interact with the microbiota and suggest that these microbial–host interactions may influence wound pain. This work also suggests potential mechanistic and therapeutic targets for further dissection of the microbial–host relationship on wound pain.

## 2 MATERIALS AND METHODS

### 2.1 Study design, setting, and sample

Study data and samples were collected as part of a multi‐aim cross‐sectional study of predictors and mechanisms of severe pain during wound dressing changes as detailed in Fiala et al.^18^ Study data and wound specimens were collected by the research team during a single study dressing change. One member of the team, a certified wound ostomy nurse (CWON), performed all study dressing changes to reduce variation in pain intensity caused by differences in technique across clinicians. Pain intensity during the dressing change was collected immediately after the dressing change was completed. All study procedures were approved by the University of Iowa Institutional Review Board. Informed consent was obtained from all participants.

Participants were recruited from the inpatient population at a large tertiary hospital in the Midwest. Inclusion criteria were 21 years of age or older and a full‐thickness open wound requiring dressings that was not of burn or diabetes‐related aetiology. Patients meeting inclusion criteria were then screened for the following exclusion criteria: (1) non‐English speaking, (2) cognitive impairment, (3) sensory impairment, (4) wound covered with necrotic/non‐viable tissue, (5) wound fistula, (6) malignancy in the wound, and (7) required debridement during the dressing. A member of the research team invited eligible patients to participate and obtained informed consent from those patients who were willing. Detailed recruitment and enrolment procedures are reported in Fiala et al.^18^ For descriptive purposes data on age, sex, and race were collected from the medical record and/or patient report. Study data were entered into research electronic data capture.^19^

### 2.2 Study variables

Study variables included severe pain during dressing change (main outcome), wound microbiome, gene expression of selected inflammatory cytokines, and selected wound factors.

#### 2.2.1 Severe pain during the dressing change

Immediately after the study dressing change, participants were asked to rate the ‘maximum’ pain experienced during the procedure on a 10‐point numeric rating scale. This scale is a valid and reliable tool for assessing acute pain intensity.^20^ We grouped pain intensity scores into categories ‘Severe’ (8‐10), ‘Moderate’ (4–7) ‘Mild’ (1–3), or ‘None’ (0). To test the relationship between severe pain and the continuous microbiome and inflammatory mediator variables, we compared means from wounds in ‘Mild’ or ‘None’ categories to those in the ‘Severe’ category.

##### Wound microbiome

The wound was cleansed with non‐bacteriostatic saline to remove surface contaminants. An Epicentre® Catch‐All™ Sample Collection was rotated over a 1 cm^2^ area near the centre of the wound free of nonviable tissue for 5 s with enough pressure to wick fluid from within the wound tissue.^21^ The swab portion was then placed in a 2 mL Eppendorf tube, capped, and placed on dry ice before storage in a −80°C freezer.^22^

After thawing, microbiome profiles were generated based on amplicon sequencing of the V1‐V3 region of the prokaryotic 16S rRNA gene. DNA was isolated from swab specimens as previously described by Kong et al.^23^ and Gardner et al.,^24^ barcoded, and sequenced in multiplex with paired‐end 300 bp chemistry on the Illumina MiSeq platform along with DNA‐free water controls for both runs, blank swab controls, and a mock community sample (Human Microbiome Project Mock Community B from BEI Resources, NIAID, NIH). Samples were split across two different sequencing runs in the order in which they were obtained.

Demultiplexed sequencing output from the 16S MiSeq runs was processed in *Qiime2* (version 2021.4) using read truncation parameters that maximised the yield of non‐chimeric read pairs from DADA2's denoising algorithm.^25^, ^26^ We combined the resulting amplicon sequence variants (ASV) tables, built an unrooted phylogeny, and assigned ASV taxonomies using the *Silva* database (version 138 SSU) and the fitted *classify*‐*sklearn* classifier.^27^ Contaminant ASVs were identified separately for each MiSeq run using the *Decontam* package, then pooled and removed from samples across runs.^28^ Read counts were aggregated at a genus level, and genera systematically over‐ or under‐represented by prevalence between the two sequencing runs were identified by *χ* ^2^ tests for proportions of binary presence/absence and removed from all samples. We then removed chloroplast and mitochondrial reads, and excluded samples with <1200 ASVs from downstream analyses, leaving 406 microbiome samples total with a mean post‐processing depth of 21107.3 reads. We analysed relative abundance as symmetric, scale‐invariant centred log‐ratio (CLR)‐transformed ASV counts, which we refer to subsequently as ‘CLR‐transformed relative abundance’ or simply ‘relative abundance’.^29^

Though we conducted our quantitative analysis and late‐stage quality control steps at a genus level, we made follow‐up estimates of the relative species compositions of reads assigned to *Staphylococcus* and *Streptococcus*, which both contain a combination of pathogenic and commensal species. We extracted all representative ASV sequences classified into either genus by Qiime2′s *q2*‐*feature*‐*classifier*. We performed *BLASTN* alignments of each sequence to the 16S ribosomal RNA database in NCBI, and classified query sequences by species if all of the following were true: a single species name was represented among the subset of the *BLASTN* hits with the highest % sequence identity to the query, the classifying database sequence had over 97% sequence identity to the query, and the classifying database sequence covered over 99% of the query sequence. We assigned ASVs to species based on the union of the *q2*‐*feature*‐*classifier* results and *BLASTN* results.

##### Gene expression of inflammatory cytokines

A second swab was obtained using the same method described above and immediately placed in 2 mL Eppendorf tube with 500 μL RNA‐Later buffer, capped, placed on dry ice before storage in a −80°C freezer.

After thawing, inflammatory mediator expression was measured by quantitative RT‐polymerase chain reaction (qPCR) on RNA extracted from swabs and converted to cDNA (Qiagen RNeasy Mini Kit, Invitrogen Superscript IV VILO kit). Amplification was performed on the ViiA7 Real‐Time PCR System with custom TaqMan array cards for 24 target genes including the 18S rRNA gene, which was used as an endogenous control (Thermo Fisher Scientific). For each gene target/sample combination, this yielded cycle threshold (CT) values, or the number of amplification cycles needed to detect a fluorescent signal.

qPCR observations with raw CT values over 35 were considered undetected and were excluded from downstream analyses. The remaining CT values were normalised to the endogenous 18S RNA control to calculate the ∆CT as (CT_Target_ – CT_18S_) for each target in each sample. Because lower ∆CT corresponds to higher levels of a target's RNA present in the sample, we analyse and report expression as (−∆CT) values. For downstream analyses, we considered only the 13 qPCR markers for which we had measures in at least 20% of all patients (*n* ≥ 89).

#### 2.2.2 Wound factors

The wound factors of wound aetiology, wound location, wound depth, wound duration, clinical inflammation, and type of wound dressing were characterised during dressing changes, following the removal of the previous dressings.

##### Wound aetiology

*Wound aetiology* was categorised as pressure ulcer, venous ulcer, surgical wound, traumatic wound, traumatic wound with surgical fix, or other. Wound aetiology was identified using the medical record or the CWON' evaluation based on differential diagnostic criteria.^30^

##### Wound location

Wound location was determined based on direct observation of the wound in relation to the participant's body. Wound location was categorised as trunk, limb, inguinal, or head/neck.

##### Wound depth

To measure depth, a swab was inserted into the wound at deepest aspect and marked at the plane of the skin. The distance from the tip of the swab to the mark was measured in centimetres.

##### Wound duration

Wound duration was defined as the time from injury/tissue loss to the time of enrolment and categorised as (A) ≤7 days; (B) 8–30 days; (C) 31–90 days; (D) 91 days to 1 year; or (E) >1 year. Although this definition has face validity, in practice its reliability is unclear, especially for chronic wounds that have been present for weeks, months, or years. To enhance validity, we crosschecked duration of injury with the medical record and the participant. We dichotomized wound duration into ≤30 days or >30 days for analyses.

##### Clinical inflammation

Clinical Inflammation was defined as the presence of erythema extending 2 cm from the wound edge in combination with heat, defined as a positive temperature gradient at the wound margin compared with a control site. Erythema was measured using an item from the Clinical Signs and Symptoms Checklist (CSSC), which was previously developed and validated by Gardner et al.^22^ The wound and control site temperatures were measured using a self‐calibrating, portable, infrared thermometric probe (Exergen Model DT 1001, Exergen Products, Watertown, MA), that measures temperature in increments of 0.1°F and is accurate to within ±0.2°F.^31^ The highest detected temperature within the perimeter of the wound was compared with the temperature at a control site that was either contralateral location or distant from the wound where the tissue appears normal (e.g., no erythema, wound, scarring). Two measures of clinical inflammation (i.e., heat, erythema, pain, and oedema) were used to increase the validity because these signs and symptoms are subtle and have low reliability when assessed individually.^30^ Clinical inflammation was dichotomized as present or absent for analyses.

##### Type of dressing

Dressings were categorised as standard (i.e., gauze‐with or without solutions such as Normal Saline or Dakins, etc.), advanced (i.e. hydrogel, negative pressure wound therapy, non‐adherent [Mepitel®, KerraContact™], alginate, or hydrocolloid), or wound VAC. Type of dressing was observed during the dressing change. Because VAC is a commonly used alternative to traditional wound dressing techniques and is thought to promote healing in multiple wound aetiologies, we also dichotomized the wound dressing type variable by the presence or absence of VAC treatment.^32^, ^33^

### 2.3 Statistical analysis methods

All statistical analyses were conducted in R, and scripts are available at https://github.com/Grice-Lab/IowaWound/. Dirichlet multinomial mixture (DMM) modelling was performed on genus‐level relative abundance tables as described in Quince et al.^34^ Associations were tested between categorical variables using chi‐squared tests and between continuous and binary variables using Wilcoxon rank‐sum tests. Correlations between inflammatory mediators and CLR‐transformed relative abundances of common genera were measured non‐parametrically using Spearman's correlation. To account for batch effects between 16S sequencing runs, we performed Wilcoxon rank‐sum tests of differences in genus relative abundance, stratified on sequencing run, using the method described in Kawaguchi and Koch.^35^ Because this is an exploratory, hypothesis‐generating study, *p*‐values are generally reported without corrections for multiple comparisons. However, significance levels after Benjamini–Hochberg corrections for the number of biological features compared with the variable of interest (e.g., 13 inflammatory markers, 5 genus relative abundance variables) are shown by asterisks in figures when indicated in figure legends.

## 3 RESULTS

### 3.1 Wound microbiome profiles were highly variable but clustered across aetiologies into four microbial community types

**FIGURE 1:**
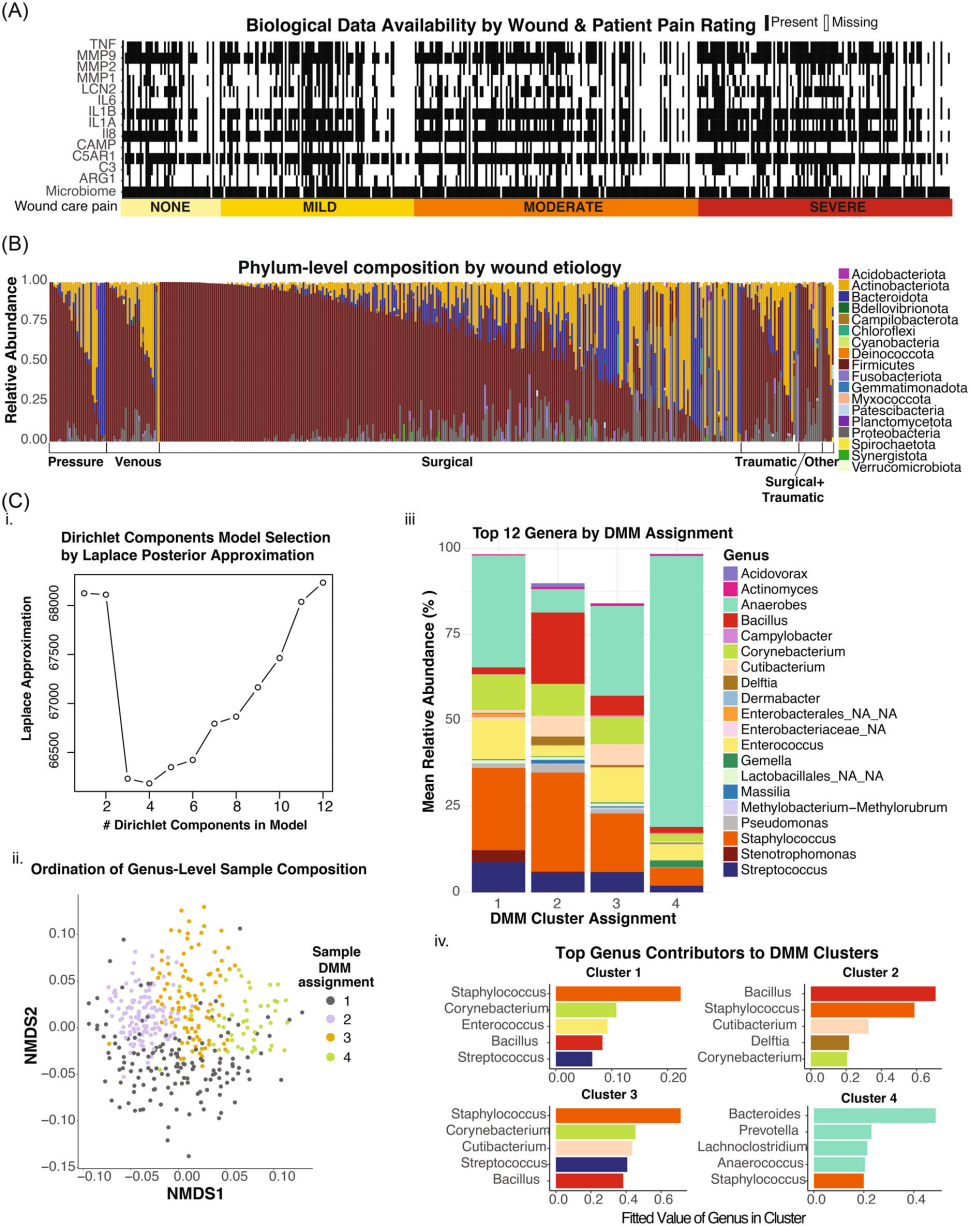
Wound microbiome variables and biological data availability. (A) Presence/absence of 13 inflammatory mediator gene expression (quantitative real‐time polymerase chain reaction) variables and microbiome profiles in each patient sample, arranged by patient‐reported pain during wound dressing change (B) Relative abundances of bacterial phyla in microbiome profiles by sample, arranged by wound aetiology. (C) Dirichlet multinomial mixture (DMM) model‐based clustering of sample microbiome profiles. (i) Laplace's approximation to posterior negative‐log evidence, for DMM models fit with between 1 and 12 mixture components (clusters) shows 4 clusters provide the optimal fit; (ii) Axes 1 and 2 for non‐metric multidimensional scaling (NMDS) ordination of sample microbiota, labelled by sample DMM cluster assignment (iii) top 12 genera by mean abundance in samples from each cluster, with strict anaerobes grouped together (iv) Mean percent relative abundance of all genera which are among the top 12 abundant genera in any cluster.

Basic demographic information and summary statistics for key patient variables are described in Table 1. In total, we analysed 16S rRNA amplicon sequencing‐based taxonomic profiles for 406 wounds (Figure 1AB). Most wounds across aetiologies were dominated by Firmicutes at the phylum level, but Actinobacteriota, Bacteroidota, and Proteobacteria were also prevalent (Figure 1B). The wound microbiome profiles were even more highly variable at a genus level, with 56 different genera dominating at least one wound by relative abundance. *Staphylococcus* was the most abundant genus in 102/406 wounds, with other common dominant genera including *Bacillus* (*n* = 48), *Corynebacterium* (*n* = 40), *Enterococcus* (*n* = 36), *Streptococcus* (*n* = 27), *Prevotella* (*n* = 23), and *Bacteroides* (*n* = 23).

**TABLE 1:** Demographics and summary statistics.

Patient/wound variables (*N* = 445)	*n* (%)
Sex	
Male	215 (48.3%)
Female	230 (51.7%)
Race (EPIC classification)	
White	419 (94.2%)
African American/Black	22 (4.9%)
American Indian/Alaska Native	2 (0.5%)
Multiracial/two or more races	1 (0.2%)
Not recorded	1 (0.2%)
Wound aetiology	
Pressure ulcer	30 (6.7%)
Venous ulcer	30 (6.7%)
Surgical wound	326 (73.3%)
Traumatic wound	33 (7.4%)
Traumatic wound + surgical fix	14 (3.2%)
Other	12 (2.7%)
Wound location	
Extremity	149 (33.5%)
Trunk	254 (57.1%)
Head/neck	5 (1.1%)
Inguinal	37 (8.3%)
Use of VAC in dressing	
VAC not used	333 (74.8%)
VAC used	112 (25.2%)
Clinical inflammation	
Inflamed	110 (24.7%)
Not inflamed	335 (75.3%)
Wound care pain	
None	54 (12.1%)
Mild	104 (23.4%)
Moderate	160 (36.0%)
Severe	127 (28.5%)
Days since injury	
≤7 days	157 (35.3%)
8–30 days	159 (35.7%)
31–90 days	66 (14.8%)
91 days‐1 year	47 (10.6%)
≥1 year	16 (3.6%)

Patient age (years)	Mean	SD	Minimum	Maximum
	57.7	14.1	21.0	95.0

Abbreviation: VAC, vacuum‐assisted wound closure.

Based on common wound inhabitants and pathogens, we selected four genera of interest to explore by relative abundance: *Corynebacterium*, which includes mostly commensal skin species, *Staphylococcus*, which includes the opportunistic skin pathogen *S*. *taphylococcus aureus* and commensals such as *Staphylococcus epidermidis*, *Pseudomonas*, which includes the wound pathogen *Pseudomonas aeruginosa*, and *Streptococcus*, which includes pathogenic and commensal species alike.^36^ Alongside these four genera, we also analysed aggregate relative abundance of strict anaerobes according to literature‐based classifications (Table S1). *Staphylococcus* was highly prevalent and abundant in our dataset, found in 83.5% of wounds overall at a mean relative abundance of 21.5%. Though both prevalent and abundant on average, anaerobes and *Staphylococcus* abundances were highly variable across samples with standard deviations of 35.7% and 32.3%, respectively. *Pseudomonas* was the rarest of our genera of interest, found in only 35/406 wounds at a mean abundance of 1.5% (Table 2).

**TABLE 2:** Microbiome and clinical variables.

Wound care pain						
		None	Mild	Moderate	Severe	
DMM cluster assignment	1	17 (32.1%)	36 (38.7%)	59 (39.9%)	35 (31.2%)	*χ* ^2^ *p* = 0.6451
	2	12 (22.6%)	28 (30.1%)	39 (26.4%)	36 (32.1%)	
	3	18 (34%)	20 (21.5%)	34 (23%)	25 (22.3%)	
	4	6 (11.3%)	9 (9.7%)	16 (10.8%)	16 (14.3%)	

Inflammation				
		Not inflamed	Inflamed	
DMM cluster assignment	1	107 (35.3%)	40 (38.8%)	*χ* ^2^ *p* = 0.4996
	2	87 (28.7%)	28 (27.2%)	
	3	70 (23.1%)	27 (26.2%)	
	4	39 (12.9%)	8 (7.8%)	

Wound duration				
		Days since injury ≤30	Days since injury >30	
DMM cluster assignment	1	99 (34.5%)	48 (40.3%)	*χ* ^2^ *p* = 0.4945
	2	83 (28.9%)	32 (26.9%)	
	3	68 (23.7%)	29 (24.4%)	
	4	37 (12.9%)	10 (8.4%)	

Wound location						
		Extremity	Trunk	Head/Neck	Inguinal	
DMM cluster assignment	1	44 (33.6%)	90 (37.7%)	2 (40%)	11 (35.5%)	*χ* ^2^ *p* = 0.0004998
	2	50 (38.2%)	59 (24.7%)	1 (20%)	5 (16.1%)	
	3	33 (25.2%)	58 (24.3%)	2 (40%)	4 (12.9%)	
	4	4 (3.1%)	32 (13.4%)	0 (0%)	11 (35.5%)	

Wound dressing practice (all aetiologies)				
		Non‐VAC	VAC	
DMM cluster assignment	1	123 (40.19%)	24 (24%)	*χ* ^2^ *p* = 0.0006562
	2	78 (25.49%)	37 (37%)	
	3	64 (20.91%)	33 (33%)	
	4	41 (13.39%)	6 (6%)	

Wound dressing practice (surgical/traumatic + surgical only)				
		Non‐VAC	VAC	
DMM cluster assignment	1	88 (40.93%)	23 (23.47%)	*χ* ^2^ *p* = 0.0001451
	2	55 (25.58%)	36 (36.73%)	
	3	39 (18.14%)	33 (33.7%)	
	4	33 (15.35%)	6 (6.12%)	

Wound aetiology								
		Pressure	Venous	Surgical	Traumatic	Traumatic + surgical	Other	
DMM cluster assignment	1	16 (55.2%)	8 (28.6%)	105 (34.9%)	9 (30%)	6 (50%)	3 (50%)	*χ* ^2^ *p* = 0.06247
	2	3 (10.3%)	8 (28.6%)	86 (28.6%)	13 (43.3%)	5 (41.7%)	0	
	3	5 (17.2%)	10 (35.7%)	71 (23.6%)	7 (23.3%)	1 (8.3%)	3 (50%)	
	4	5 (17.2%)	2 (7.1%)	39 (13%)	1 (3.3%)	0 (0%)	0	

Common wound genus abundances				
Genus‐level classification	Mean (%)	Median (%)	Standard deviation (%)	No. of positive samples at >0.1%
*Anaerobes*	29.07%	8.46%	35.7%	340
*Corynebacterium*	8.48%	0.434%	19.41%	264
*Pseudomonas*	1.46%	0%	9.67%	35
*Staphylococcus*	21.47%	4.11%	32.27%	339
*Streptococcus*	6.43%	0.228%	17.77%	224

Abbreviations: DMM, Dirichlet multinomial mixture; VAC, vacuum‐assisted wound closure.

To classify the genus‐level microbiome profiles into microbial community types, we clustered the 406 genus‐level microbiome profiles using DMM modelling.^34^ Fitting the DMM model with between 1 and 12 components showed that four components yielded the optimal Laplace approximation of fit (Figure 1C.i). Based on the four‐component model, we assigned each sample to the DMM cluster whose corresponding model it had the highest probability of being generated from (Figure 1C.ii). Clusters 1, 2, and 3 were dominated by *Staphylococcus* along with other contributing genera such as *Bacillus*, *Corynebacterium*, *Enterococcus*, and *Cutibacterium*. Cluster 4, which contained only 47 (11.6% of the total) samples, was dominated almost entirely by anaerobic genera, which were aggregated for visualisation and interpretation following clustering (Figure 1C.iii,iv). DMM cluster distribution did not statistically differ between aetiologies or patient‐reported wound care pain but did differ by wound location and dressing type (*χ* ^2^ *p* = 0.0005 and 0.0007, respectively, Table 2). Clusters 3 and 4 showed higher average genus‐level diversity, by both richness and Shannon diversity metrics, than clusters 1 and 2 (Figure S1).

### 3.2 Host inflammatory gene expression is associated with clinical inflammation but not patient‐reported pain

**FIGURE 2:**
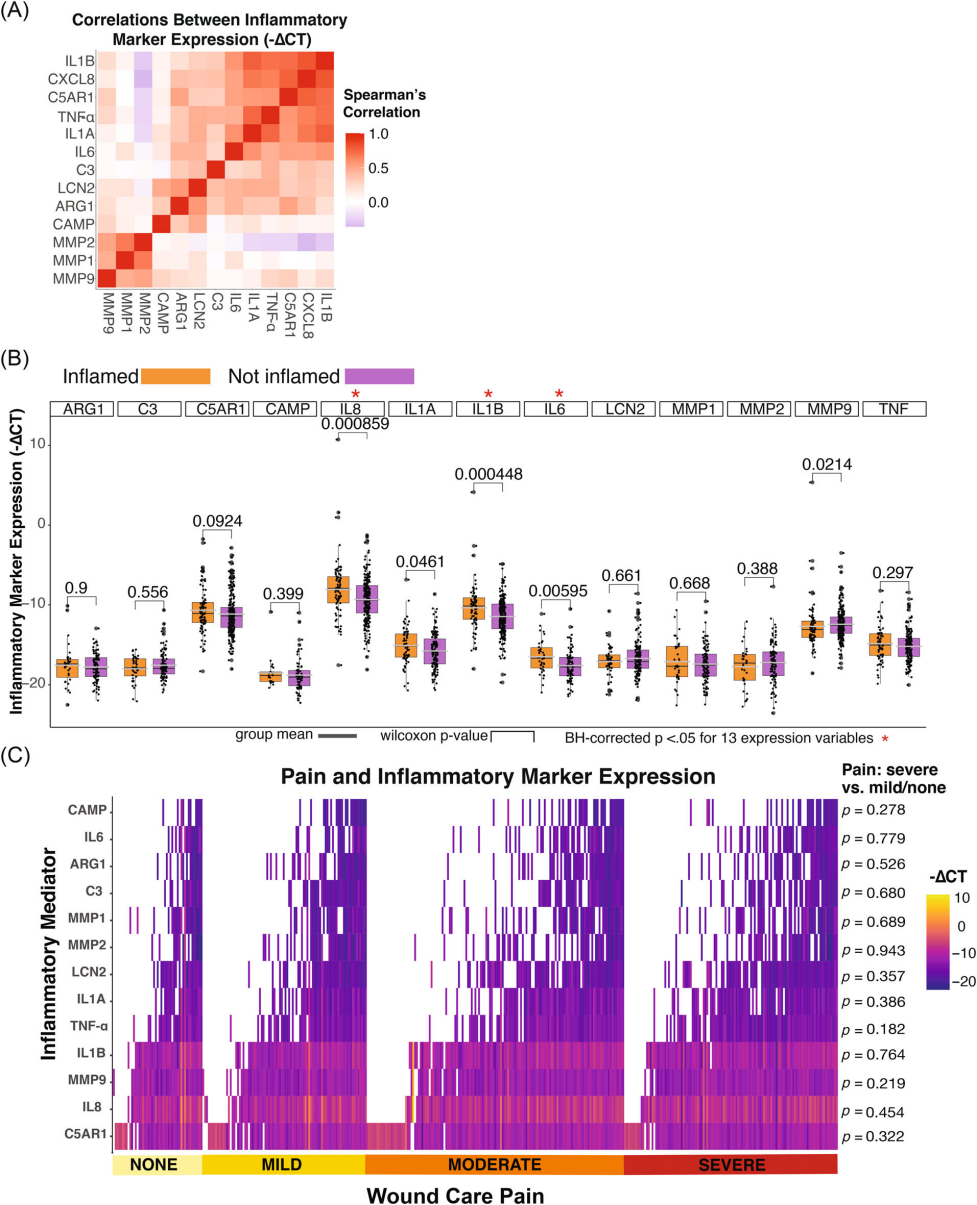
Host inflammatory gene expression is strongly associated with clinical inflammation but is not associated with patient‐reported pain. (A) Pairwise Spearman correlations between inflammatory mediator gene expression variables (−∆CT value), arranged by the results of Euclidean hierarchical clustering of the correlation distance matrix. (B) Boxplot comparisons of inflammatory mediator expression in samples from clinically inflamed (left, orange) versus not inflamed (right, purple) wounds; *p*‐values are Wilcoxon rank sum test *p*‐values, and red asterisks indicate corrected *p* < 0.05 by Benjamini–Hochberg FDR procedure for 13 comparisons. (C) Heatmap showing inflammatory mediator gene expression (−∆CT) by sample, grouped by patient‐reported pain category for wound dressing change. For each inflammatory mediator, *p*‐values are reported from a Wilcoxon rank sum test for a difference in mean expression between wound samples from patients who reported no or mild pain versus severe pain during dressing changes.

We hypothesized that genes involved in inflammation may be predictive of patient‐reported pain during WCP and would be associated with other host and microbial factors in the wound environment. After excluding undetected samples and gene variables missing in >80% of samples, we examined expression of 13 different genes (Figure 1A). Based on hierarchical clustering of correlations between expression, we found that inflammatory marker genes grouped together consistently with established inflammatory pathways (Figure 2A). For example, the complement component five receptor C5AR1, which mediates the activation of inflammatory responses by neutrophils and other immune cells, was strongly co‐expressed with the pro‐inflammatory cytokines IL‐1α, IL‐1β, IL‐6, and tumour necrosis factor‐α (TNF‐α), along with the chemoattractant cytokine IL‐8, which recruits neutrophils to wounds in response to inflammation.^37^, ^38^ The matrix metalloproteinases MMP2, MMP1, and MMP9 also clustered together by expression, consistent with their coordinated role in extracellular matrix remodelling during wound healing.^39^ Arginase 1 (ARG1) and Lipocalin 2 (LCN2) are involved in the innate immune response and were co‐expressed with the gene encoding cathelicidin antimicrobial peptide (CAMP), which responds to signals of inflammation (Figure 2A).^40^, ^41^, ^42^, ^43^ Additionally, IL‐8, IL‐1β, and IL‐6 genes were more highly expressed in wounds with clinically demonstrable inflammation (Figure 2B).

Despite exhibiting biologically and clinically consistent signals among observations meeting our minimum expression thresholds, none of the inflammatory mediators tested were significantly associated with patient‐reported wound care pain (Wilcoxon rank‐sum *p*‐values >0.18, Figure 2C). This could be in part due to the overall sparsity of the inflammatory mediator data owing to low RNA yields from wound samples. For example, LCN2 and IL‐6, which have both previously been linked to neuropathic pain were only measured at detectable levels in 46% and 27% of samples, respectively (Figure 1A).^44^, ^45^ The relationship between these inflammatory mediators and pain during wound dressing change, therefore, remains uncertain. It is also possible that other wound characteristics, such as time since injury and location, influence relationships between inflammatory gene expression and patient‐reported pain. Among just wounds which are >30 days old, for example, IL‐1B expression is higher on average in wounds with severe pain than those with mild or no pain (Figure S2, *p* = 0.00596).

### 3.3 The abundance of common wound genera is associated with patient‐reported pain and host inflammatory gene expression

**FIGURE 3:**
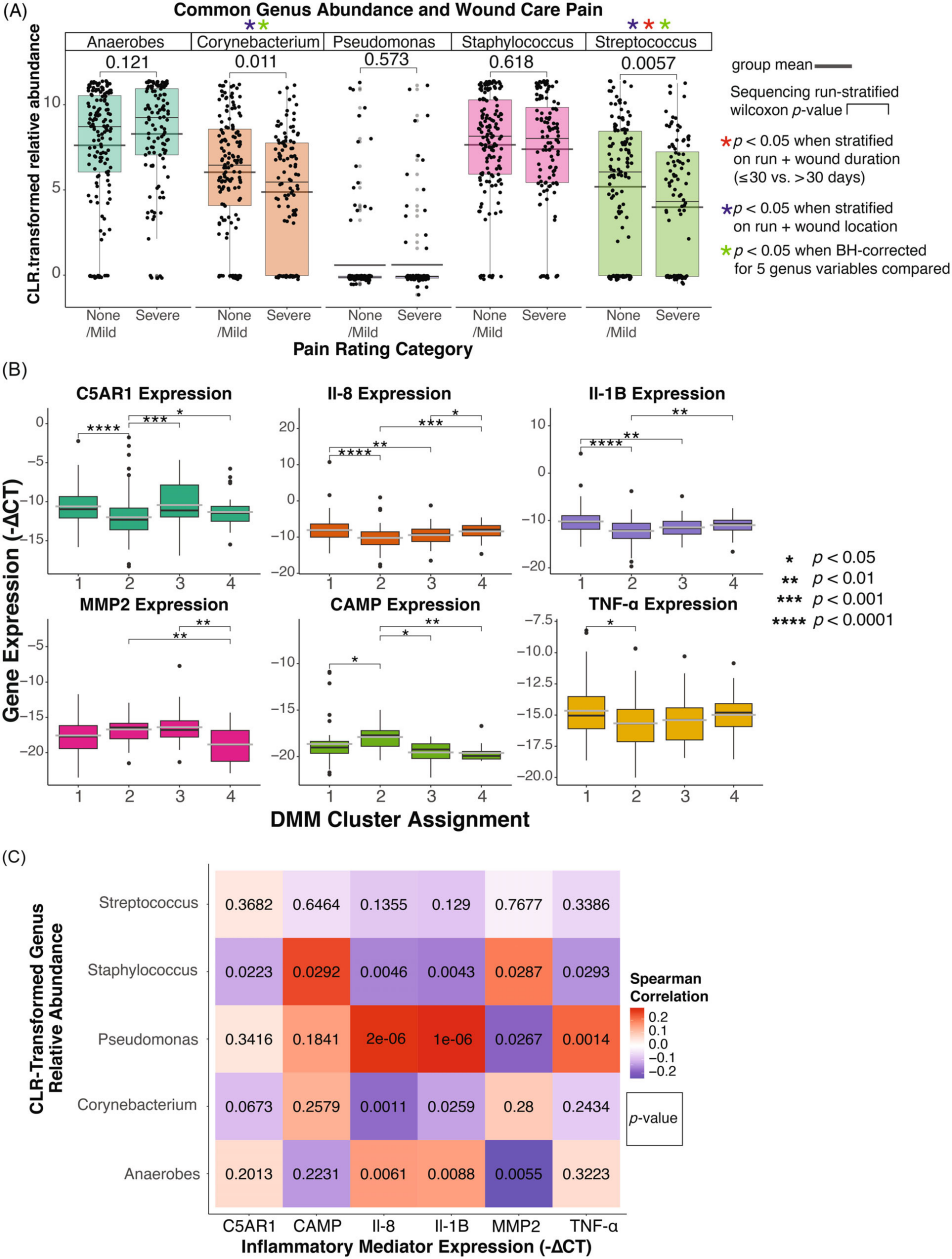
Relationship between the microbiome and patient factors. (A) Boxplots of common wound genera abundance (centred log‐ratio [CLR]‐transformed) in wounds where patients reported none/mild (left) versus severe pain (right) during dressing changes. *pp*‐values for a Wilcoxon rank sum test of difference in means, stratified by sequencing run, are shown. Comparisons which remain significant after a Benjamini–Hochberg FDR correction for five genus comparisons are indicated with a green asterisk; Comparisons which remain significant after stratifying on wound duration in addition to sequencing run are indicated with a red asterisk; Comparisons which remain significant after stratifying on wound location are indicated with a dark blue asterisk (B) Boxplots of relative expression (−∆CT) of six inflammatory mediators in samples assigned to each of the four Dirichlet multinomial mixture (DMM) clusters, with significant Wilcoxon rank sum *pp*‐values highlighted for pairwise comparisons for inflammatory mediator means between clusters. (C) Pairwise Spearman's rank correlations between six inflammatory mediators' expression (*x*‐axis) and CLR (*y*‐axis).

Though we did not find a direct association between inflammatory mediator expression and patient‐reported pain during wound care, we did find a relationship between several key wound and skin inhabitants and this clinical outcome. Relative abundances of *Corynebacterium* (stratified Wilcoxon *p* = 0.011) and *Streptococcus* (*p* = 0.0057) were lower in samples from patients reporting severe wound care pain than in those from patients reporting mild pain or no pain (Figure 3A).^46^ However, we also identified that *Corynebacterium* was of higher abundance in wounds over 30 days of age than wounds which had been open for 30 days or less (Figure S3A), and that patients with ≤30 day‐old wounds were more likely to report severe pain during dressing changes (52.0%) than patients with older wounds (25.9%). When we further stratified the comparisons of key genera relative abundances between wounds with and without severe pain by wound duration (≤30 days or >30 days), *Streptococcus* remained negatively associated with severe pain ratings (*p* = 0.00238), while *Corynebacterium* did not (*p* = 0.136, Figure S3B). Wound duration could simultaneously contribute to higher *Corynebacterium* abundance and lower incidence of severe wound pain, confounding *Corynebacterium*'s relationship to pain. We found no such relationship between *Streptococcus* and days since injury, suggesting that the lower *Streptococcus* in wounds causing severe pain is not explained by wound duration. Both *Corynebacterium* (*p* = 0.0185) and *Streptococcus* (*p* = 0.0200) remained negatively associated with severe pain ratings after stratifying for wound location (extremity, trunk, head/neck, or inguinal, Figure S3C). When stratified on a combination of sequencing run, wound duration, and location together, *Streptococcus* also remained negatively associated with severe pain ratings (*p* = 0.0153) while *Corynebacterium* did not (*p* = 0.209).

Six different inflammatory mediators were associated with DMM microbiome cluster: complement receptor C5AR1 (Kruskal–Wallis *p* = 1.11e−05), chemokine IL‐8 (*p* = 3.93e−07), inflammatory cytokine IL‐1β (*p* = 7.34e−07), matrix metalloproteinase MMP2 (*p* = 0.0101), antimicrobial peptide CAMP (*p* = 0.0194), and TNF‐α (*p* = 0.0354; Figure 3B). In follow‐up pairwise comparisons, C5AR1, IL‐8, and IL‐1β were expressed more highly in cluster 1 samples than cluster 2 or 3 samples. Cluster 2 samples, defined by lower anaerobic genera abundance and high abundances of *Bacillus* and *Staphylococcus*, expressed lower levels of these three genes than samples in the other clusters. MMP2, which was negatively correlated with multiple pro‐inflammatory genes, was expressed at the lowest levels in cluster 4 samples, which are marked by a predomination of anaerobic genera. TNF‐α was most highly expressed in cluster 1 samples, while CAMP was most highly expressed in cluster 2 samples, which were dominated by *Bacillus* and *Staphylococcus*.

To further examine the relationship between the microbiome and these six inflammatory mediators, we examined non‐parametric rank correlation between their expression and the five genus abundance variables of interest (Figures 3C and S4). Interestingly, *Staphylococcus* and *Corynebacterium* relative abundance were both negatively correlated with the expression of genes in the pro‐inflammatory cluster, and *Staphylococcus* abundance was positively correlated with MMP2 expression. Conversely, anaerobe abundance and *Pseudomonas* abundance were correlated with IL‐8 and IL‐1β but negatively correlated with MMP2. CAMP was positively correlated with *Staphylococcus*, while TNF‐α was negatively correlated with *Staphylococcus* but positively correlated with *Pseudomonas*.

Though we conducted our V1–V3 16S analyses at a genus‐level, the *Streptococcus* and *Staphylococcus* genera contain pathogenic and commensal organisms alike. We conducted a follow‐up analysis to roughly estimate the proportions of species constituting the reads belonging to each genus. In *Staphylococcus*, *S*. *epidermidis* was of the highest relative proportion on average within samples (49.8%) followed by still‐unclassified *Staphylococcus* (17.2%) and *S*. *aureus* (6.01%). In *Streptococcus*, unclassified *Streptococcus* reads made up the highest relative proportion on average (14.9%), followed by *Streptococcus thermophilus* (5.90%) and *Streptococcus salivarius* (4.78%; Figure S6).

### 3.4 VAC is associated with higher levels of *Staphylococcus* and lower levels of anaerobes

**FIGURE 4:**
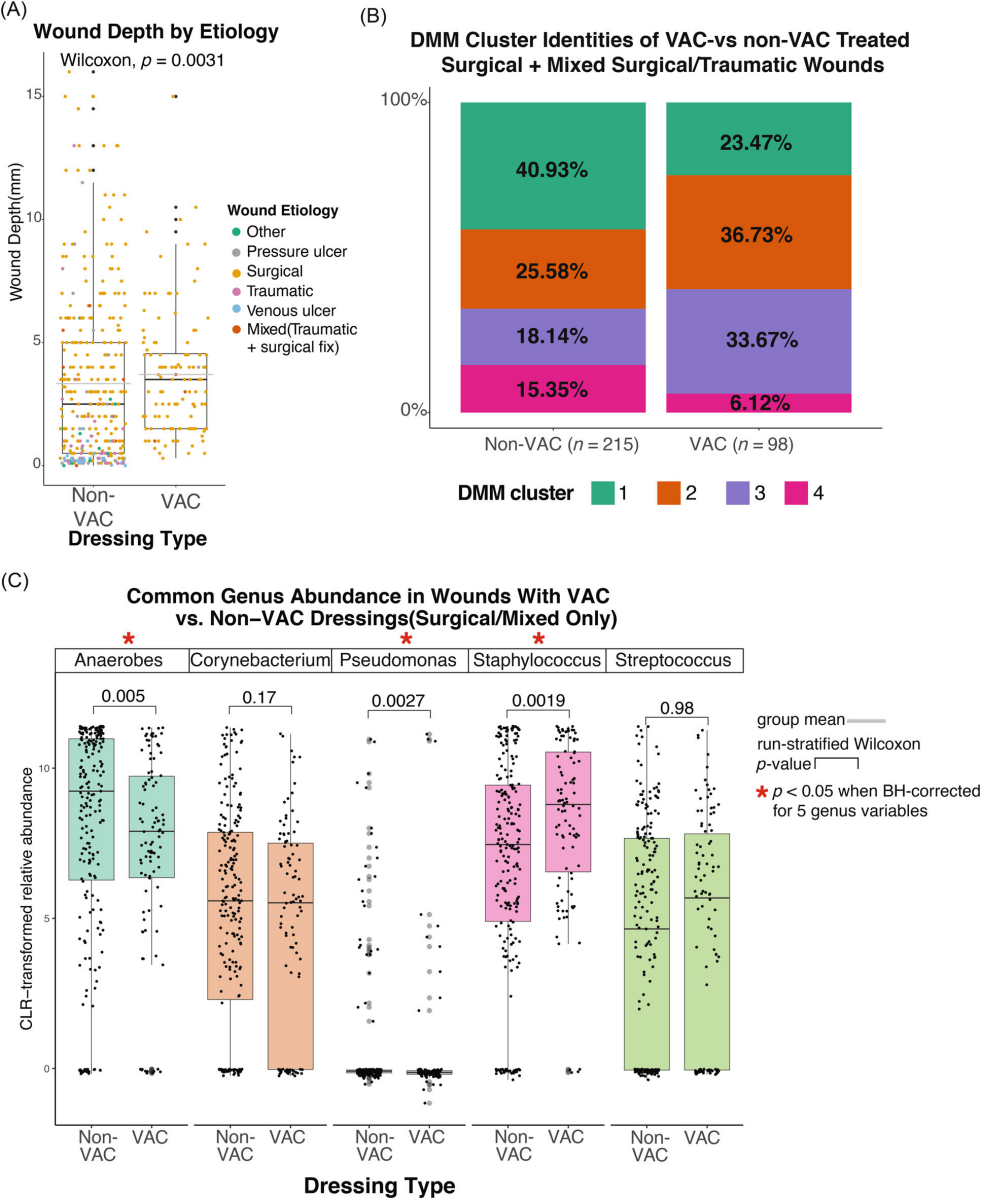
Relationships between dressing types and the wound microbiome (A) Boxplots of wound depth (*y*‐axis) versus the use (right) or absence (right) of vacuum‐assisted closure (VAC) by aetiology, showing an association by stratified Wilcoxon signed rank test between VAC use and wound depth which is largely driven by aetiology. (B) Bar plot showing proportions of non‐VAC treated (left) and VAC‐treated (right) wound microbiome samples assigned to each of the four Dirichlet multinomial mixture (DMM) clusters (C) Boxplots of common genus relative abundance in wounds treated with (right) and without (left) vacuum‐assisted closure.

We observed a non‐random distribution of DMM microbiome clusters by wound dressings applied with and without VAC (Table 2). However, we also noted that use of VAC was almost entirely limited to surgical wounds and traumatic wounds with surgical fixation. Of the samples with corresponding microbiome profiles, 98 of 313 wounds of these two aetiologies underwent VAC, compared with only 2 out of 93 wounds of other origins (Figure 4A). We therefore limited our analysis of associations between VAC and microbiome variables to the 313 wounds of ‘surgical’ and ‘traumatic with surgical fix’ aetiologies. Within this grouping, we found that VAC‐treated wound microbiomes were less commonly assigned to DMM clusters 4 or 1, and more often assigned to clusters 2 or 3 than microbiome profiles from non‐VAC‐treated wounds (Figure 4B and Table 2). At a genus level, we observed higher abundances of *Staphylococcus* (stratified Wilcoxon *p* = 0.0019) and lower abundances of anaerobic genera (*p* = 0.005) in VAC‐treated than non‐VAC‐treated wounds, as well as lower abundances of *Pseudomonas* (*p* = 0.0027; Figure 4C).

We considered wound depth as a possible confounder of these relationships since VAC is more often used on deeper wounds. Consistent with our previous findings in a diabetic foot ulcer context, we found wound depth to be positively correlated with anaerobic genus abundance (*p* = 8.77e−06, Spearman's *r* = 0.219) and negatively correlated with *Staphylococcus* abundance (*p* = 5.36 × 10^−8^, Spearman's *r* = −0.266).^24^ Within the 313 surgical or combined surgical/traumatic wounds, however, we observed no difference in wound depth between VAC‐ and non‐VAC‐treated wounds (Wilcoxon *p* = 0.5122), meaning that depth is not likely to confound our observations within this wound aetiology. We conclude, therefore, that VAC‐treated wounds had significantly higher relative abundances of *Staphylococcus* and lower relative abundances of strictly anaerobic genera on average.

Having observed a relationship between these two genus abundance variables and VAC treatment, we considered that the observed relationships between pain and two other genus variables, *Corynebacterium* and *Streptococcus* abundance, could be confounded by VAC use. However, we observed that both *Streptococcus* (*p* = 0.008) and *Corynebacterium* (*p* = 0.033) were both still significantly lower in wounds with severe pain ratings than those with mild or no pain after stratifying for VAC use (Figure S5A). When we compared genus abundance between wounds with severe and mild or no reported pain in a more homogeneous (albeit less well‐powered) subset of the data, trunk wounds which were not treated with VAC, *Corynebacterium* (*p* = 0.024) was significantly lower in severe wounds in this subset, while *Streptococcus* (*p* = 0.093) was also lower, but not significantly so.

## 4 DISCUSSION

To our knowledge, this study represents the most comprehensive exploration of relationships between wound microbiome composition, patient factors, and severe pain during dressing change procedures to date. By comparing 16S amplicon sequencing profiles to wound factors and quantified inflammatory mediator expression in 406 wounds, we sought to identify novel microbial and gene expression markers of severe pain during wound dressing changes.

Despite not finding a relationship between severe wound care pain and overall microbial composition as measured by DMM community assignment, we found that both *Corynebacterium* and *Streptococcus* were of higher abundance in wounds of patients who reported no pain or mild pain compared with those who reported severe pain during their procedure. Methodologically, this reflects the complementary nature of these two lines of analysis: while DMM clustering by taxonomic read distribution enabled us to look at large‐scale differences in composition between samples, targeted analysis of four common wound genera and the aggregated obligate anaerobes enabled us to uncover potential interaction between specific taxa of interest and clinical factors.

The inverse relationship between *Corynebacterium* abundance and severe wound pain is consistent with a genus containing species, which primarily act as commensals on the human skin and which lack the toxigenic properties of common pathogens.^47^ However, the observed relationship between *Corynebacterium* abundance and pain was not significant after stratifying for wound duration, suggesting decreased rates of severe pain and increased *Corynebacterium* abundance in older wounds may explain this relationship rather than *Corynebacterium's* own effect on pain.

*Streptococcus*, on the other hand, remained negatively associated with severe pain after stratifying on either location, wound duration, or both variables. Our finding that *Streptococcus* abundance is negatively correlated with severe pain is surprising given the ability of *S*. *pyogenes* to induce pain during necrotizing fasciitis infection via secreted streptolysin S's activation of pain receptors. While *S*. *pyogenes* is an alpha haemolytic pathogen which regularly causes wound infections, the healthy skin is also commonly colonised by *Streptococcus* species which more commonly act as commensals, such as *Streptococcus pseudopneumoniae* and *Streptococcus mitis*.^48^, ^49^, ^50^ Though the highest proportion of *Streptococcus* reads per sample on average were unclassified at a species level even after our BLAST‐based follow‐up analysis, only 0.74% of *Streptococcus* reads per sample on average were classified as *S*. *pyogenes* (Figure S6). The seemingly contradictory relationship we observed between *Streptococcus* and severe pain, therefore, could owe to the presence of non‐pathogenic *Streptococcus* which lack the toxic activity of *S*. *pyogenes*. Interestingly, Choi et al.^36^ identified *Streptococcus* and *Corynebacterium* as being lower in chronic wounds of patients with autoimmune diseases.

We were also surprised to find that none of the 13 inflammatory gene markers we tested were associated with severe wound care pain, particularly given several markers' correlations with clinical inflammation and previous evidence that inflammation is a key contributor to pain following injury.^51^, ^52^ However, this finding is consistent with the fact that we did not observe a higher rate of severe pain as compared with mild or no pain in patients with inflamed wounds than those without (*χ* ^2^ *p* = 0.3488).

We did observe a relationship between several key inflammatory mediators and the wound microbiome. Anaerobes and *Pseudomonas*, whose abundances were correlated, were positively associated with the pro‐inflammatory C5AR1, IL8, and IL‐1β but inversely correlated with the anti‐inflammatory MMP2. *Staphylococcus* and *Corynebacterium* showed the opposite relationship, corresponding to lower levels of the pro‐inflammatory factors. This provides evidence for the relationship between common wound genera and the inflammatory immune response. This is contrary to previous findings that infection with the opportunistic pathogen *S*. *aureus* stimulates IL‐8 production in nasal and intestinal epithelial cells, and activates C5AR1 to stimulate the production of IL‐1β in blood.^53^, ^54^, ^55^ However, like *Streptococcus*, *Staphylococcus* encompasses a wide range of skin‐ and wound‐associated commensal organisms in addition to *S*. *aureus* which have species‐ and even intraspecies strain‐dependent effects on the cutaneous immune response.^56^ In our BLASTN follow‐up analysis of *Staphylococcus*‐classified reads, we classified just under 50% of *Staphylococcus* reads within samples on average as belonging to *S*. *epidermidis*, which is commonly considered to be a commensal skin species. Conversely, the commonly studied wound pathogen, *S*. *aureus*, made up only 6.1% of these reads, while 17.2% remained unclassified.

In the process of examining the relationship between patient factors and the wound microbiome, we found a relationship between microbial composition and the use of VAC in wounds of surgical and mixed traumatic/surgical aetiology. VAC works by applying negative pressure to the wound, which stabilises the wound bed, clears wound exudate and contaminant materials, and increases blood flow.^32^, ^33^, ^57^, ^58^ VAC has previously been shown to reduce the rate of surgical site infections compared with traditional dressings and is thought to reduce overall bioburden, but evidence on effects on specific pathogens, including *S*. *aureus* and *P*. *aeruginosa*, is conflicting.^57^, ^59^, ^60^ Overall microbial composition, as measured by DMM community type assignment, was different between VAC‐treated and non‐VAC‐treated wounds, with VAC‐treated wounds less likely to be assigned DMM microbial community type 1 or 4, and more likely to be assigned type 2 or 3 than non‐VAC treated wounds (Table 2). Consistent with this result, we also found that strict anaerobes were of lower abundance, but *Staphylococcus* was of higher abundance, in VAC‐treated surgical wounds as compared with those untreated with VAC. Though we cannot conclude a directional relationship between our variables due to the observational nature of this study, our findings motivate further investigation into the effects of VAC on wound microbiome composition.

There are several methodological limitations to this study. First, while V1V3 16S rRNA amplicon sequencing is an efficient, high‐throughput method of profiling the wound microbiome, its reliance on a sub‐region of a single gene makes it difficult to confidently infer taxonomic assignments at a greater than genus‐level resolution for many taxa. The skin, and especially wounds, are low bacterial biomass sites dominated by human cells, which makes it difficult to get comprehensive samples of the wound microbiota with a non‐invasive swabbing technique. Similarly, these swabs yielded variable amounts of human RNA for qPCR measures of inflammatory gene marker expression, resulting in sparse measures of the markers across the 445 patients, which likely reduced some of our power to detect relationships between the inflammatory mediators and pain (Figure 1A). Finally, this is an exploratory, observational study performed at one collection point per patient. Though its design enables the direct sampling of patient wounds from a variety of different sources and pathologies, it precludes us from assessing causal relationships between the observed variables.

In summary, we present evidence that the wound microbiome is highly variable across aetiologies, but correlates with inflammatory host gene expression, patient‐related pain, and VAC treatment. These findings from 406 wounds motivate more targeted studies to further refine specific associations, as well as mechanistic studies to establish direction of causation and shed light on additional targetable pathways.

## CONFLICT OF INTEREST STATEMENT

The authors declare no conflicts of interest.

## Supporting information


**Table S1.** Patient and wound description.

**Figure S1.** Microbiome variation across 406 wound samples. (A) Principal coordinates analysis on weighted Unifrac distances between genus‐aggregated microbiome samples, with PERMANOVA conducted on sequencing run showing variation between samples sequenced in different runs. (B) Genus richness (top) and Shannon diversity (bottom) of samples in each DMM cluster, where *p*‐values are from a Wilcoxon signed rank test (C) Relative abundance (proportion) of any genera which are the top‐12 most abundant in any DMM cluster, by sample, grouped by cluster assignment. (D) Relative abundance (proportion) of any genera which are the top‐12 most abundant in any DMM cluster, by sample, grouped by wound aetiology and labelled by DMM cluster assignment.
**Figure S2.** Inflammatory mediator expression and wound care pain in subsets of wounds ≤30 days (top) and >30 days (bottom). Gene expression values (−∆CT) are shown with Wilcoxon *p*‐values in differences between pain groups are shown. Note: none of these comparisons met *p* < 0.05 threshold after a Benjamini–Hochberg correction for 13 inflammatory mediators compared.
**Figure S3.** Relationships between key genera abundances and patient factors. (A) Relative abundances of five genus variables in wounds ≤30 days old (left) or >30 days old (right). (B) Relative abundances of five genus variables in wounds with severe (right) versus none/mild (left) pain ratings, stratified by wound duration to ≤30 days (top) or >30 days (bottom). (C) Relative abundances of five genus variables in wounds with severe (right) versus none/mild (left) pain ratings, stratified by wound location to extremity (top), trunk (middle), inguinal (bottom), or head/neck (not shown due to absence of mild/none ratings in this stratum).
**Figure S4.** Relationships between key genera abundances and inflammatory mediators Scatterplots of key genera abundances versus gene expression (shown as correlation heatmaps in Figure 3C).
**Figure S5.** Relationships between genus abundance and severe pain after stratifying for VAC use. (A) Relative abundances of five genus variables in wounds with severe (right) versus none/mild (left) pain ratings, stratified by the presence or absence of VAC treatment (B) Relative abundances of five genus variables in wounds with severe (right) versus none/mild (left) pain ratings in just trunk wounds without VAC treatment.
**Figure S6.** Estimated species classifications of *Staphylococcus* and *Streptococcus* reads. As described in Section 3.3, we performed a BLASTN alignment of *Staphylococcus*‐ and *Streptococcus*‐classified 16S V1V3 sequencing reads to classify reads into species. NA refers to reads which could not be classified, and the plot of estimated relative proportions of *Streptococcus* (top) and *Staphylococcus* (bottom) species are ordered by the abundances of the top three classifications by mean proportion. The visualisation is limited to species classifications which made up at least 5% of ASVs in at least one sample.

## Data Availability

The data that support the findings of this study are available from the corresponding author upon reasonable request.
