# Supplementary figures and images for: The heterogeneous wound microbiome varies with wound care pain, dressing type, and inflammatory gene expression
Source: Wound Repair Regen. 2024 Apr 26;32(6):811–25. doi: 10.1111/wrr.13184 (PMC11511792; doi:10.1111/wrr.13184)

Figure S1.

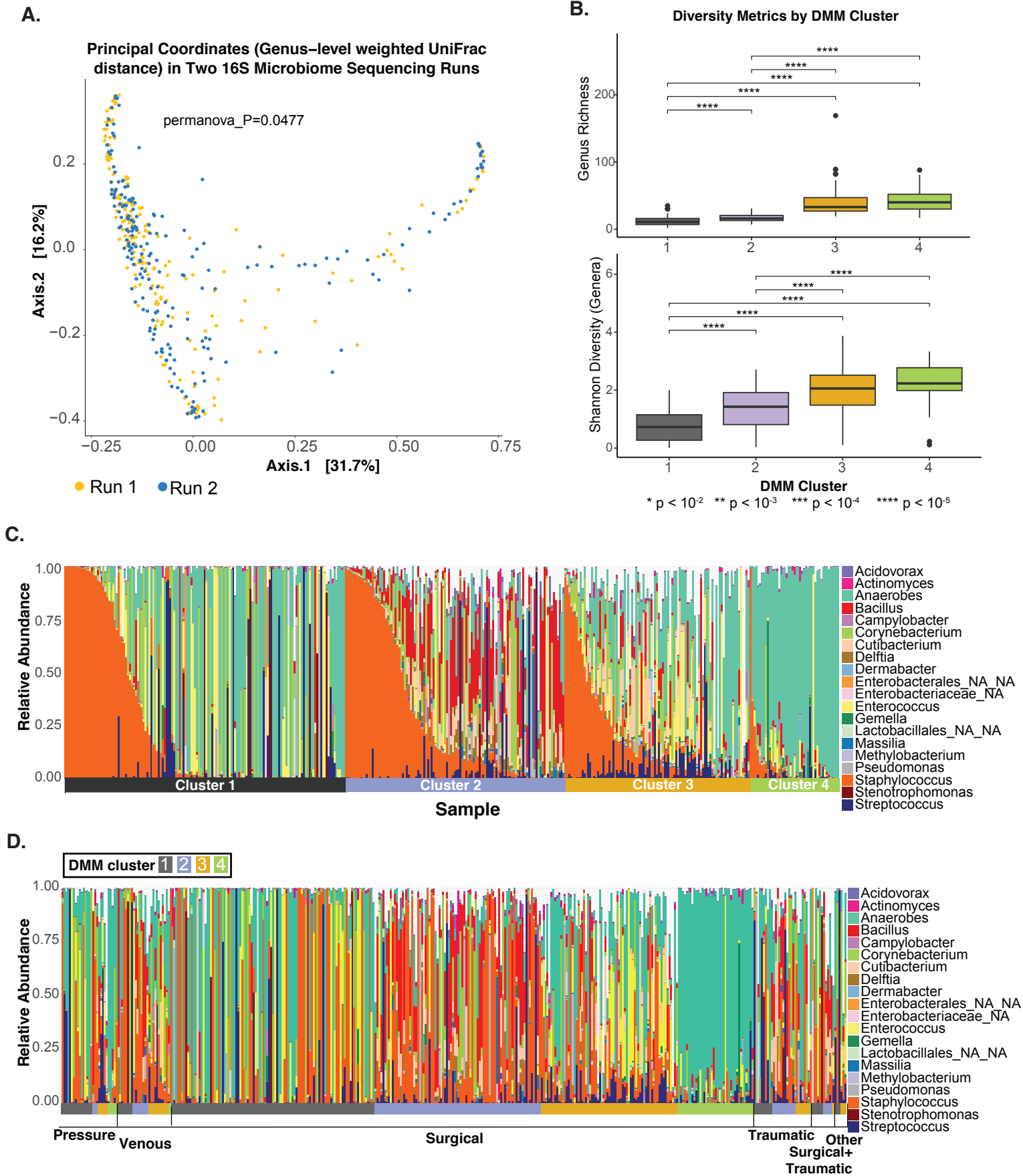

**Figure S2.**

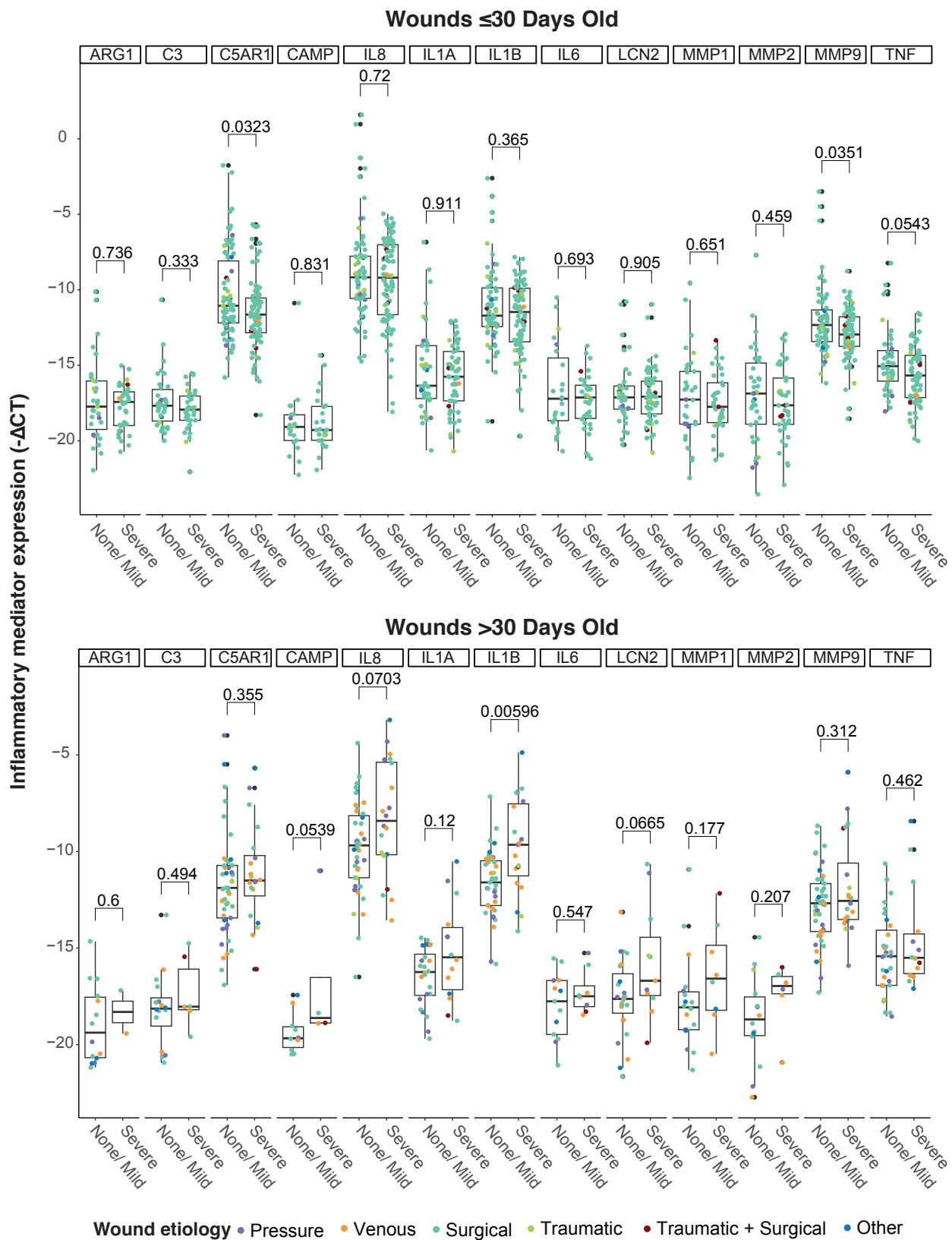

Figure S3

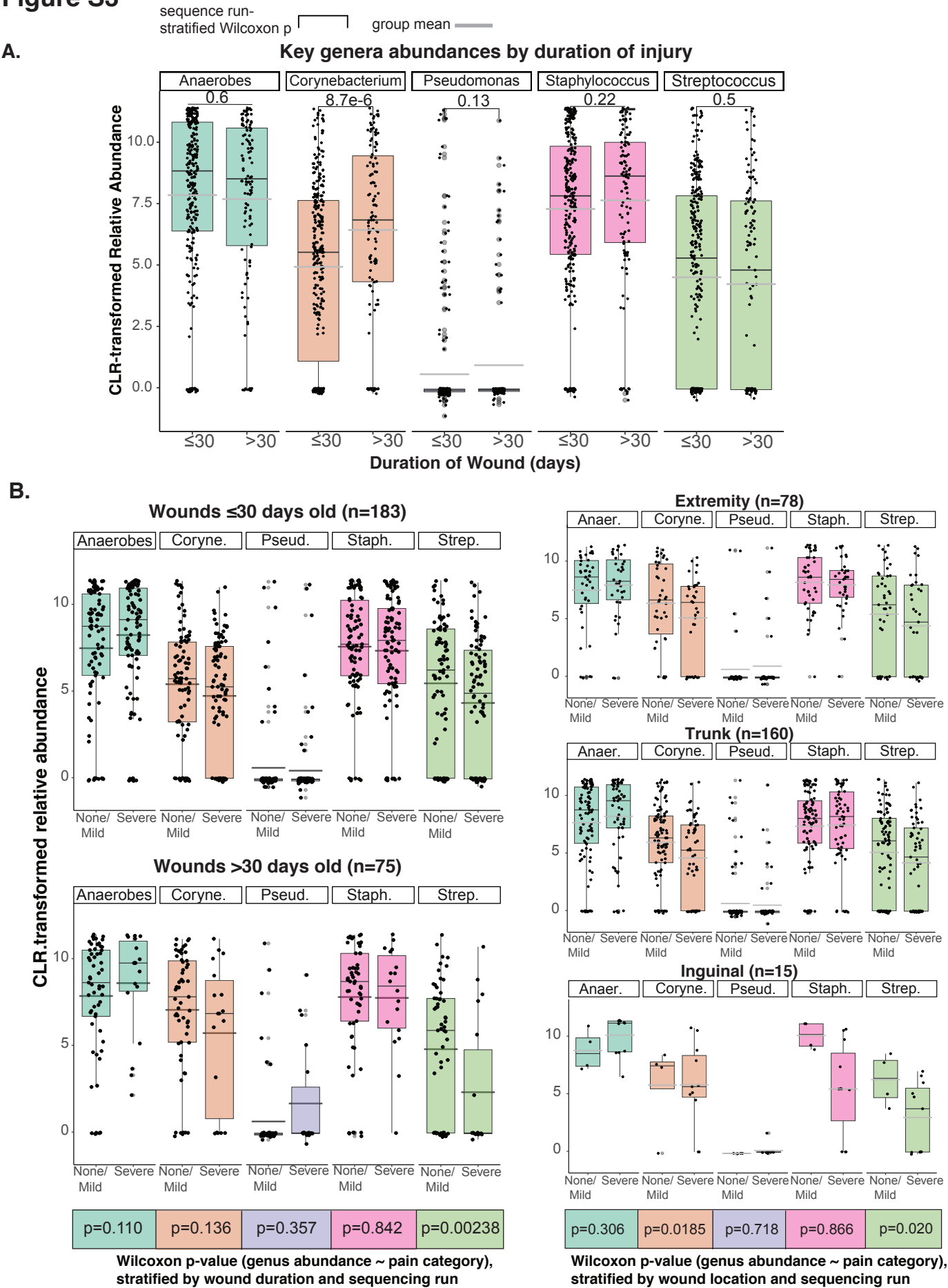

Figure S4.

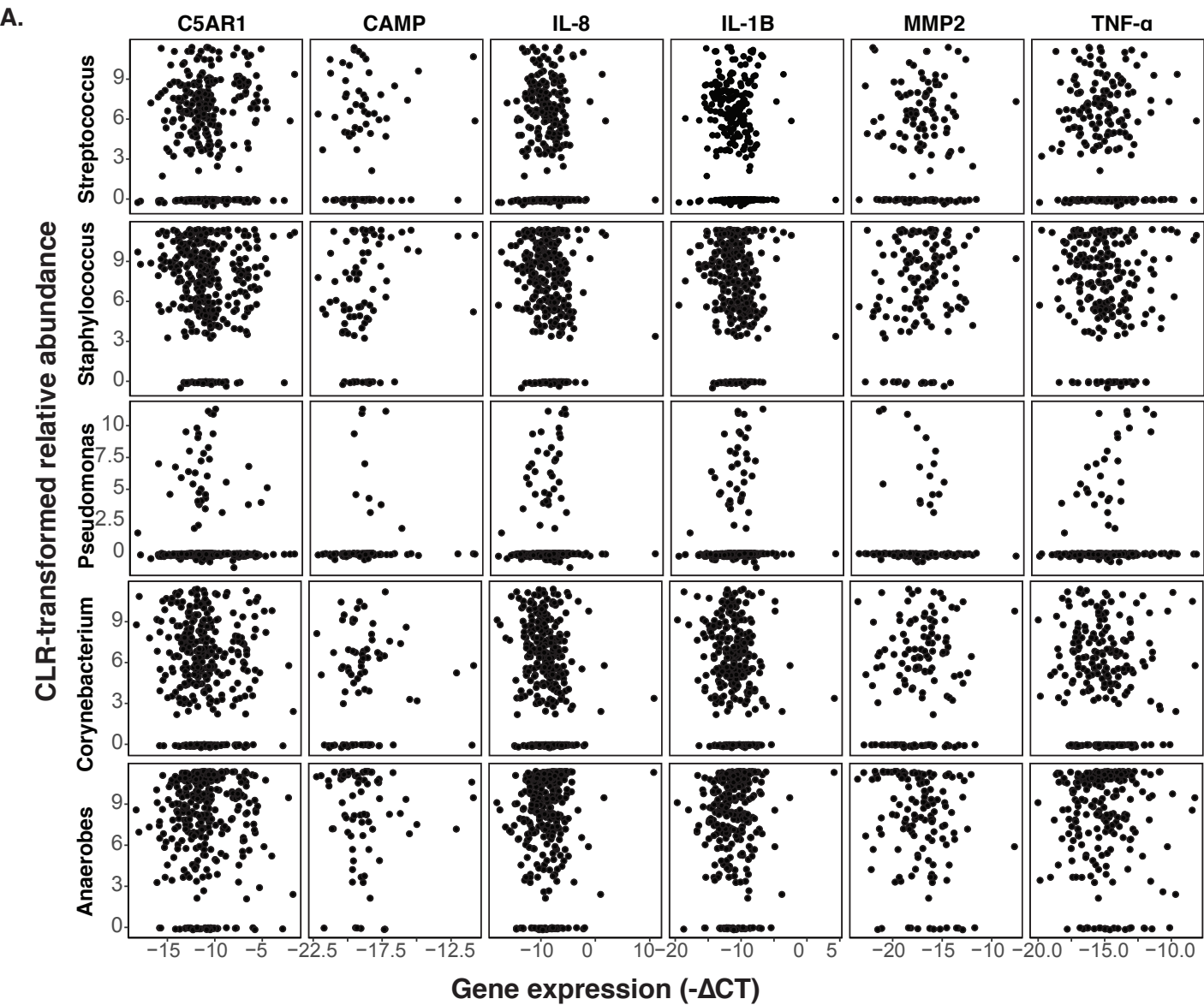

Figure S5.

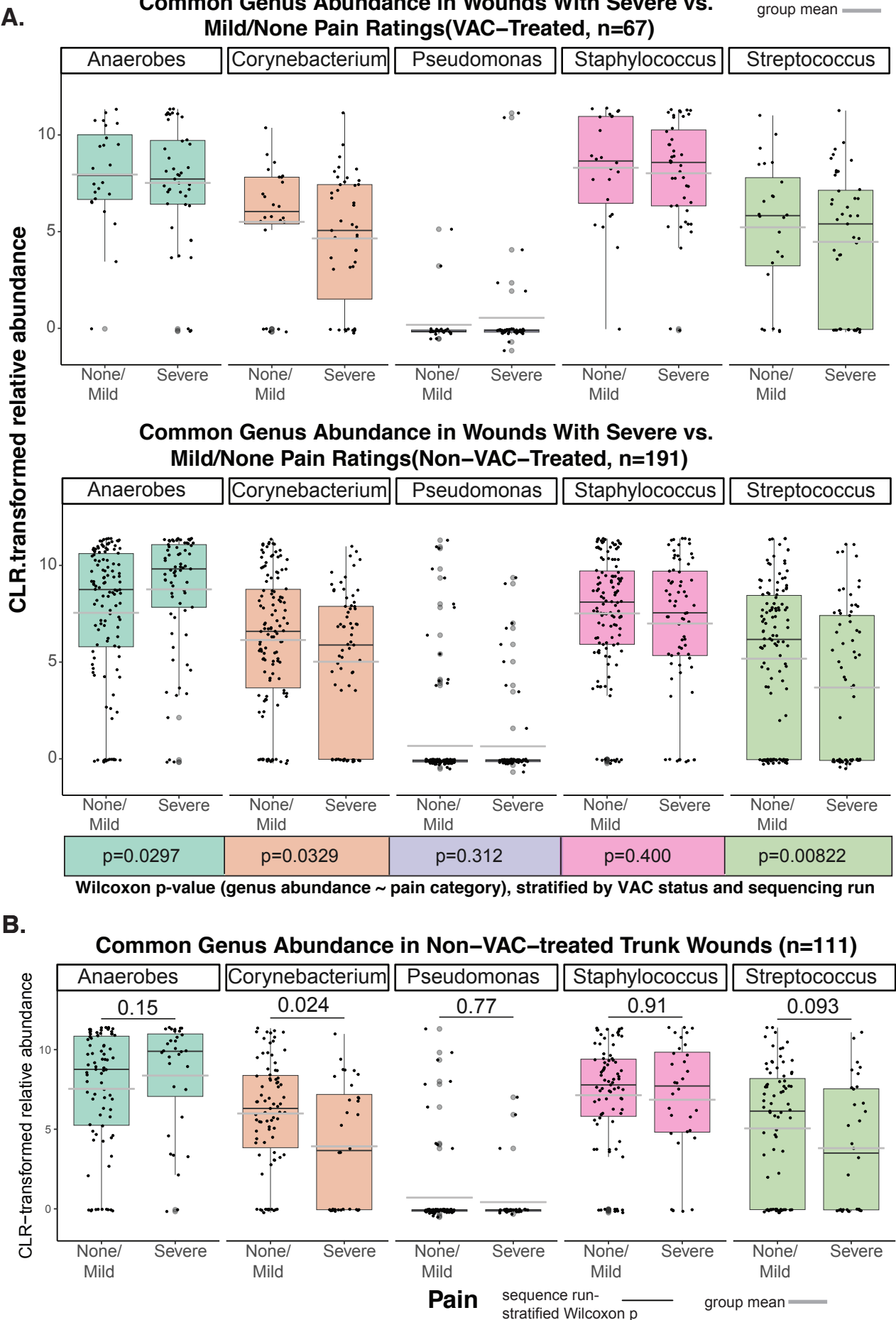

Figure S6.

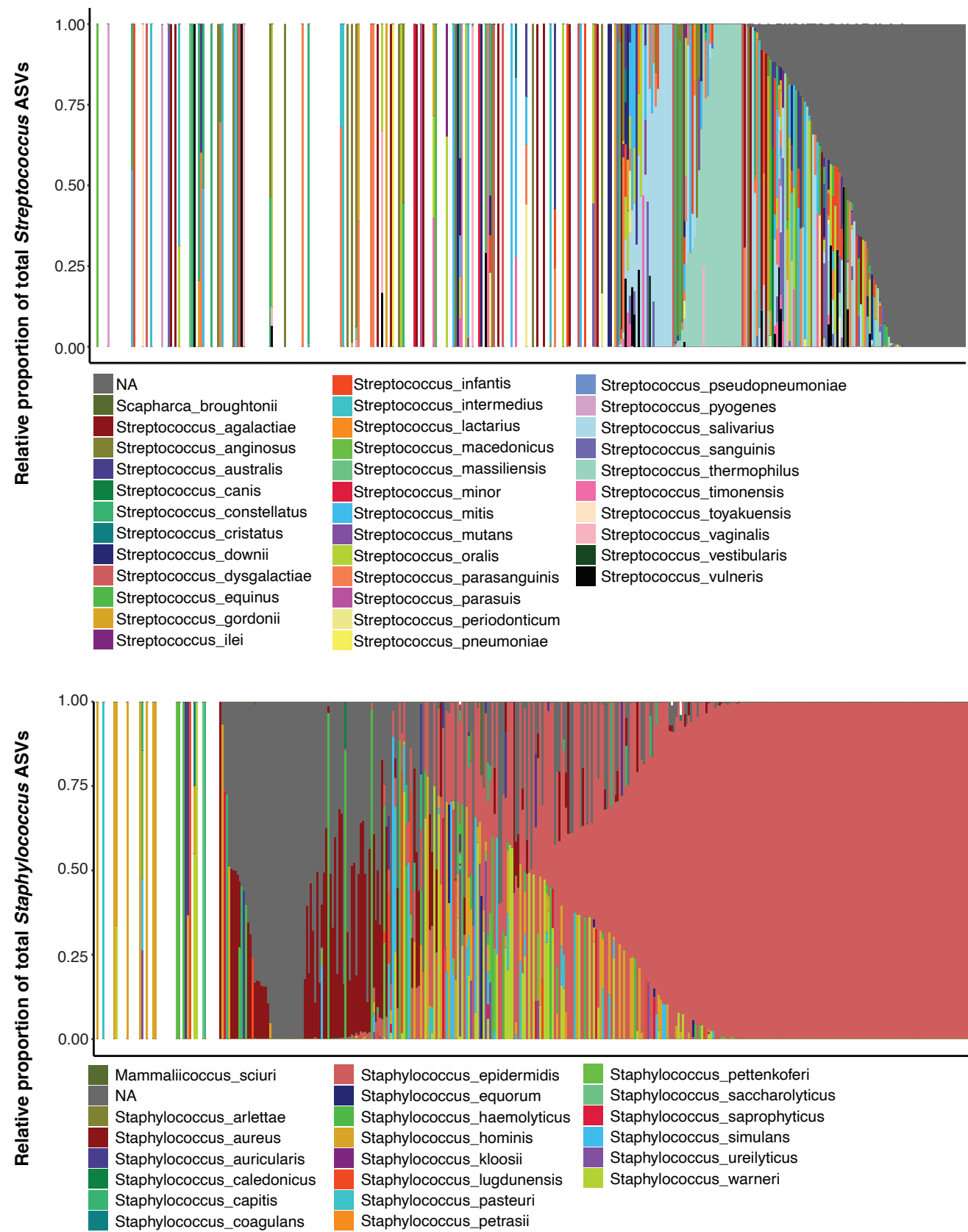

Supplement: Supplementary file 2 — Figure S1. Microbiome variation across 406 wound samples. (A) Principal coordinates analysis on weighted Unifrac distances between genus‐aggregated microbiome samples, with PERMANOVA conducted on sequencing run showing variation between samples sequenced in different runs. (B) Genus richness (top) and Shannon diversity (bottom) of samples in each DMM cluster, where p‐values are from a Wilcoxon signed rank test (C) Relative abundance (proportion) of any genera which are the top‐12 most abundant in any DMM cluster, by sample, grouped by cluster assignment. (D) Relative abundance (proportion) of any genera which are the top‐12 most abundant in any DMM cluster, by sample, grouped by wound aetiology and labelled by DMM cluster assignment. Figure S2. Inflammatory mediator expression and wound care pain in subsets of wounds ≤30 days (top) and >30 days (bottom). Gene expression values (−∆CT) are shown with Wilcoxon p‐values in differences between pain groups are shown. Note: none of these comparisons met p < 0.05 threshold after a Benjamini–Hochberg correction for 13 inflammatory mediators compared. Figure S3. Relationships between key genera abundances and patient factors. (A) Relative abundances of five genus variables in wounds ≤30 days old (left) or >30 days old (right). (B) Relative abundances of five genus variables in wounds with severe (right) versus none/mild (left) pain ratings, stratified by wound duration to ≤30 days (top) or >30 days (bottom). (C) Relative abundances of five genus variables in wounds with severe (right) versus none/mild (left) pain ratings, stratified by wound location to extremity (top), trunk (middle), inguinal (bottom), or head/neck (not shown due to absence of mild/none ratings in this stratum). Figure S4. Relationships between key genera abundances and inflammatory mediators Scatterplots of key genera abundances versus gene expression (shown as correlation heatmaps in Figure 3C). Figure S5. Relationships between genus abundance and severe [file WRR-32-811-s001.pdf]
